# Green Synthesized Silver Nanoparticles Induced Accumulation of Biomass and Secondary Metabolites in Hairy Roots of *Rehmannia glutinosa*

**DOI:** 10.3390/ijms252313088

**Published:** 2024-12-05

**Authors:** Yunhao Zhu, Xiangxiang Hu, Le Dong, Han Yang, Danning Zhou, Xiangnan Liu, Chengming Dong, Xiule Yue, Le Zhao

**Affiliations:** 1 School of Pharmacy, Henan University of Traditional Chinese Medicine, Zhengzhou 450046, China; guxinhan123@163.com (Y.Z.); 15837616094@163.com (X.H.); 15890596169@163.com (L.D.); 18339537995@163.com (H.Y.); 17837337981@163.com (D.Z.); 15515670901@163.com (X.L.); dcm371@sohu.com (C.D.); 2 Ministry of Education Key Laboratory of Cell Activities and Stress Adaptations, School of Life Sciences, Lanzhou University, Lanzhou 730000, China

**Keywords:** hairy roots, secondary metabolites, silver nanoparticles, *Rehmannia glutinosa*

## Abstract

The hairy roots of *Rehmannia glutinosa* (Gaertn.) Libosch. ex Fisch. & C. A. Mey. are capable of producing active compounds such as iridoid glycoside, and phenylethanoid glycosides, which have potential applications in the pharmaceutical industry. Silver nanoparticles (AgNPs) have been used as novel elicitors in the induced cultivation of hairy roots, but there is a lack of research regarding their effects on *R. glutinosa* hairy roots. In the present study, silver nanoparticles (Pp-AgNPs) synthesized by the endophytic fungus *Penicillium polandii* PG21 were adopted to elicit hairy roots of *R. glutinosa*, to investigate their influences on the biomass, color, secondary metabolites, antioxidant activity, sucrose metabolism, and phytohormone-related gene expression. The results revealed that the dry weight and fresh weight of *R. glutinosa* hairy roots were both higher in the treated group than in the control group after addition of 2 mg/L Pp-AgNPs for 20 d. The content of verbascoside, total phenylethanol glycosides and total cycloartenoid in the treatment group reached the highest level at 20 d, which were 1.75, 1.51, 1.44 times more than those in the control group, respectively. Pp-AgNPs significantly stimulated the enzyme activities of catalase (CAT), superoxide dismutase (SOD), and peroxidase (POD). The growth-promoting effect of Pp-AgNPs may be accomplished by increasing sucrose metabolism, and regulating the synthesis and signal transduction of gibberellin (GA) and indoleacetic acid (IAA). Moreover, expressed sequence tags-simple sequence repeat (EST-SSR)-based genetic diversity analyses indicated that there was little possibility of genetic variation among samples under different treatment conditions. In conclusion, the appropriate concentration of Pp-AgNPs can be used as an effective elicitor to improve the biomass and secondary metabolites content in *R. glutinosa* hairy roots.

## 1. Introduction

Hairy roots are not only rapid-growing and genetically stable, but also capable of synthesizing the secondary metabolites in the original plant, which makes them suitable for in vitro culture. Therefore, the application of hairy root culture can provide an effective way to solve the shortage of plant resources. At present, reports on hairy roots mainly concentrate on the production of active ingredients in medicinal plants, such as terpenoids (glycyrrhizic acid) [1], phenolic acids (rosemarinic acid) [2], flavonoids (rutin) [3], alkaloids (camptothecin, vinblastine, atropine) [4], saponins (ginsenosides) [5], and polysaccharides [6], etc., and the hairy root cultivation system has been successfully established in dozens of medicinal plants. In recent years, the hairy roots of several medicinal plants, including *Astragalus membranaceus* (Fisch.) Bunge [7], *Lithospermum erythrorhizon* Siebold & Zucc. [8], *Panax ginseng* C. A. Mey. [9], and *Catharanthus roseus* (L.) G. Don [10], have been cultivated on a large scale.

The content of secondary metabolites in hairy roots is generally low and far from the requirements for industrial production. The usage of elicitors has emerged as one of the most effective methods to enhance secondary metabolites production in plant cell and tissue culture [11]. During plant secondary metabolism, certain biotic elicitors (fungi, bacteria, viruses, yeast, and plant cell wall extracts) and abiotic elicitors (methyl jasmonate, salicylic acid, heavy metals, and rare earth elements) can be employed as specific stress signals to activate the expression of specific genes in a rapid, specific, and selective manner, thereby regulating the biosynthesis of secondary metabolites in plant cells [12].

Metallic nanoparticles (NPs) play an important role in the production of plant secondary metabolites, which is largely attributed to their unique properties such as a small size and large specific surface area [13]. The utilization of NPs has the potential to regulate plant growth and improve plant resistance under stress conditions. Previous studies have demonstrated that NPs can coordinate the balance between plant growth and resistance by stimulating reactive oxygen species (ROS) production and triggering systemic acquired resistance (SAR) responses. Furthermore, NPs are capable of impacting plant secondary metabolism through regulating ROS yield and the expression of genes involved in secondary metabolism biosynthesis. Consequently, NPs have also been employed as abiotic elicitors in the induced culture of hairy roots [14]. For example, the biosynthesis of taxol in *Taxus wallichiana* Zucc. was significantly enhanced by the application of gold nanoparticles (AuNPs) [15]. Similarly, the biosynthesis of stevioside in *Stevia rebaudiana* (Bertoni) Bertoni was stimulated by the usage of titanium dioxide nanoparticles (TiO_2_NPs) [16], while the application of manganese trioxide nanoparticles (Mn_2_O_3_NPs) resulted in an increase in phenolic, flavonoid, and alkaloid content in *Atropa belladonna* L. stem tip cultures [17].

In recent years, silver nanoparticles (AgNPs), as a novel class of abiotic elicitors, have been applied in the induced cultivation of hairy roots, which not only promote biomass accumulation of hairy roots, but also improve the production of secondary metabolites in a variety of hairy roots [18]. For instance, the treatment with 10 μg/mL of AgNPs was able to enhance biomass accumulation of *Celastrus paniculatus* Willd. hairy roots, as well as the content of celastrol, which was observed to be 2.24-fold higher in the hairy roots [19]. The application of AgNPs resulted in 2.42 times increase in atropine content in the hairy roots of *Datura metel* L., after 48 h of treatment [20]. Artemisinin content in *Artemisia annua* L. hairy roots induced by 900 mg/L Ag-SiO_2_ NPs enhanced significantly from 1.67 mg/g to 2.86 mg/g [21].

*Rehmannia glutinosa* is a well-known traditional Chinese medicine, the root of which is utilized as a medicinal herb. The main secondary metabolites in *R. glutinosa* are iridoid glycoside and phenylethanoid glycosides, which have a number of important pharmacological effects, including hypoglycemic, improvement of the central and blood system, and antitumor effects [22]. Piatczak et al. [23] found that the hairy roots of *R. glutinosa* were capable of accumulating iridoid glycoside (including loganin and catalposide), and phenylethanoid glycosides (e.g., verbascoside and isoverbascoside) [23]. The hairy roots may serve as a candidate to produce secondary metabolites in *R. glutinosa*, which could have potential applications in the pharmaceutical industry. Studies on elicitors of *R. glutinosa* hairy roots have predominantly focused on phytohormone, including methyl jasmonate (MeJA) and salicylic acid (SA) [24,25], while the usage of AgNPs as elicitors in hairy roots culture of *R. glutinosa* has not been reported. In our previous study, we synthesized AgNPs with a particle size of approximately 3–25 nm utilizing the endophytic fungus *Penicillium polandii* PG21, which was named Pp-AgNPs [26]. Based on the above research, the present study aims to analyze the effects of different concentrations of Pp-AgNPs on the growth and secondary metabolites in the hairy roots of *R. glutinosa*. This will facilitate the exploration of methods to effectively raise the content of secondary metabolites in *R. glutinosa* hairy roots through the application of Pp-AgNPs. Moreover, this study provides a foundation and a novel valid approach for the industrial production of *R. glutinosa* hairy roots.

## 2. Results

### 2.1. Effect of Pp-AgNPs on the Growth of R. glutinosa Hairy Roots

**Figure 1:**
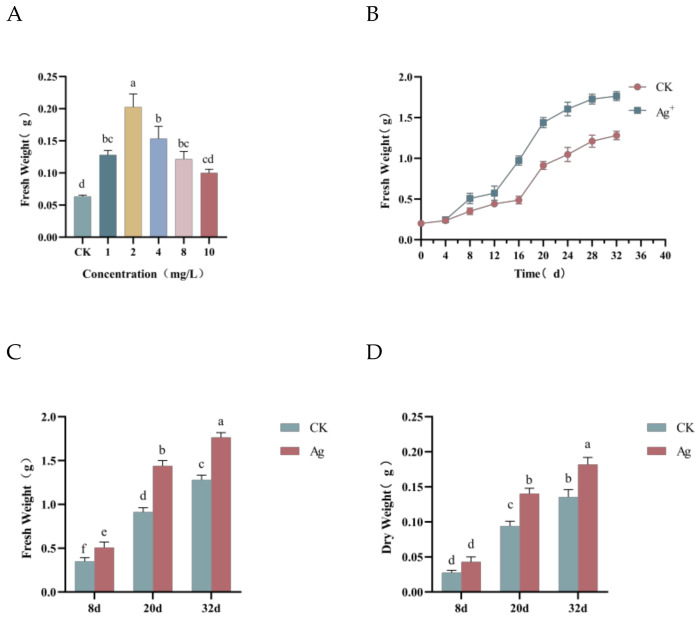
Effect of Pp-AgNPs on the growth of *R. glutinosa* hairy roots. (**A**) Different concentration treatments with Pp-AgNPs. (**B**) Growth curve. (**C**) Fresh weight of *R. glutinosa* hairy roots treated with 2 mg/L of Pp-AgNPs. (**D**) Dry weight of *R. glutinosa* hairy roots treated with 2 mg/L of Pp-AgNPs. Different letters represent statistical significance (*p* < 0.05).

When the concentration of Pp-AgNPs was greater than 10 mg/L, the hairy roots in the medium died and could not grow normally, whereas the fresh weights (FW) of hairy roots in the treatment groups were all significantly higher than those of the control group when the Pp-AgNPs concentration was less than 10 mg/L (1–10 mg/L), which exhibited a certain growth-promoting effect (Figure 1A). When the Pp-AgNPs concentration was 2 mg/L, the fresh weight of hairy roots was the highest, so that 2 mg/L was preliminarily confirmed as the appropriate concentration for subsequent studies.

The hairy roots were induced with 2 mg/L of Pp-AgNPs to construct the growth curve, and the results are shown in Figure 1B, with an S-shaped growth curve. The hairy roots were in a period of stagnation for the first 8 d, with slow growth and insignificant changes of the fresh weight. After 12 d of cultivation, the hairy roots entered the logarithmic growth phase, with vigorous cell viability, an accelerated growth rate, and a rapid increase in fresh weight, of which the fastest growth rate was observed at 20 d. The hairy roots entered the stabilization plateau phase after 24 d. As the incubation time extended, the nutrients in the medium became gradually insufficient, the growth of hairy root slowed down, and finally a stable period was reached. At this time (32 d), the fresh weight of the treatment group was 1.764 ± 0.05 g, which was 1.38 times that of the control group. Overall, the growth status of hairy roots in the treatment group was remarkably better than that of the control group and reached the rapid growth period preferentially.

The hairy roots were treated with 2 mg/L of Pp-AgNPs, and the samples were collected at 8 d, 20 d, and 32 d to determine the dry and fresh weights. The results indicated that the biomass of the treatment group was significantly better than the control group, which was 1.15, 1.47, and 1.37 times higher than the control, respectively (Figure 1C). The trend of dry weight results was similar to that of fresh weight, with the treatment group being 1.49 and 1.35 times more than the control group, respectively (Figure 1D). These results revealed that the exogenous addition of Pp-AgNPs can effectively prompt the accumulation of the hairy root biomass of *R. glutinosa.*

### 2.2. Accumulation of Ag in the Hairy Roots of R. glutinosa

After exogenous supplementation of Pp-AgNPs, there was a dramatic increase in the treatment group compared with the control group at all periods in the hairy roots of *R. glutinosa*. In the control group, the Ag content remained at an extremely low level, whereas in the treatment group, it did not improve with the prolongation of the incubation time, but showed a high-low-high trend (Table 1). This may indicate that the hairy roots have a certain saturation threshold for the uptake of AgNPs.

### 2.3. Color Alteration of R. glutinosa Hairy Roots Treated with Pp-AgNPs

The colorimeter was used to detect the powder color of the control and treatment groups at three growth stages of hairy roots, and the differences in color were represented by L*, a*, b* values. L* indicated object brightness; a* indicated object red-green; b* indicated object yellow-blue.

**Figure 2:**
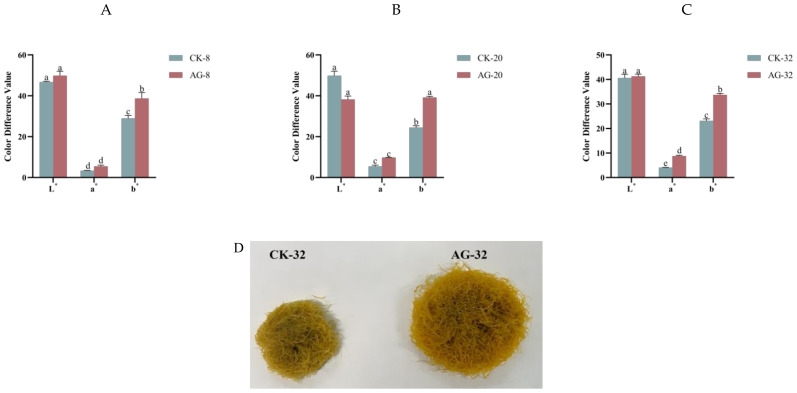
Color alteration of *R. glutinosa* hairy roots after Pp-AgNPs treatment. (**A**) Pp-AgNPs treated for 8 d. (**B**) Pp-AgNPs treated for 20 d. (**C**) Pp-AgNPs treated for 32 d. (**D**) Color changes in hairy roots after 32 d of treatment with Pp-AgNPs. Different letters represent statistical significance (*p* < 0.05).

The difference in L* and a* values between the control and treatment groups was not statistically significant, but there was a dramatic difference in b* values, indicating that the color of the hairy roots in the Pp-AgNPs-treated group was yellower compared to the control group (Figure 2). These results demonstrated that the addition of Pp-AgNPs resulted in a notable yellowish color of the hairy roots. Xue et al. [27] reported that there was a correlation between the color of the *R. glutinosa* root cross-sections and the content of chemical constituents, with L* and b* values positively correlating with the content of some components. The smaller the b* value, the lower the catalpol and martynoside D content, while the larger the b* value, the higher the martynoside D content. Therefore, we hypothesized that the color of the hairy root powder altered after the addition of Pp-AgNPs, suggesting its chemical composition changed as well, where the increase in the b* value may represent an enhancement in the content of iridoid glycosides, such as martynoside D.

### 2.4. Pp-AgNPs Treatment Raised Antioxidant Enzyme Activities and H_2_O_2_ Content in R. glutinosa Hairy Roots

**Figure 3:**
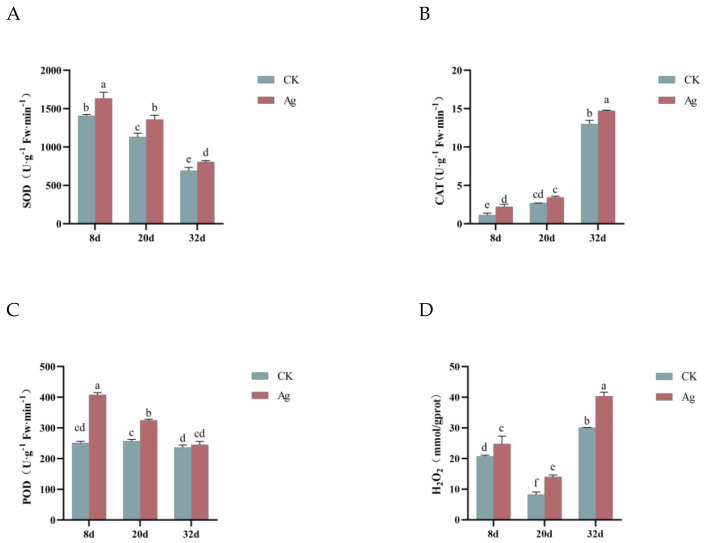
Effect of Pp-AgNPs on antioxidant enzyme activities and H_2_O_2_ content in *R. glutinosa* hairy roots. (**A**) SOD. (**B**) CAT. (**C**) POD. (**D**) H_2_O_2_ content. Different letters represent statistical significance (*p* < 0.05).

When plants are stressed by AgNPs, they will generate large amounts of reactive oxygen species (ROS), which trigger oxidative stress. As one of the representative molecules of ROS, hydrogen peroxide (H_2_O_2_) is also a signal molecule in plants, which plays an important role in response to adversity, disease prevention, and resistance, as well as regulation of growth and development. In the present study, after the treatment with Pp-AgNPs, the H_2_O_2_ content in the hairy roots obviously improved at three growth phases. In the early stage (8 d), the H_2_O_2_ content of the treatment group was 1.29 times more than that of the control group (Figure 3D). With the extension of the incubation (20 d), the H_2_O_2_ content, although decreased, was still higher than the control. At the late period of the growth (32 d), the H_2_O_2_ content of the treatment group was the highest (Figure 3D).

As important antioxidant enzymes in plants, superoxide dismutase (SOD), catalase (CAT), and peroxidase (POD) are capable of eliminating ROS species and play essential protective roles in plant growth and development and adaptation to the environment. In this study, we found that Pp-AgNPs significantly stimulated the enzyme activities of CAT, SOD, and POD, and the activities of all three antioxidant enzymes were remarkably increased compared with the control group after Pp-AgNPs treatment at different times (Figure 3A–C).

### 2.5. The Accumulation of Secondary Metabolites in R. glutinosa After Pp-AgNPs Addition

Catalpol and martynoside D could not be detected in the hairy roots of *R. glutinosa* [23]. After treatment with Pp-AgNPs, the verbascoside content in the control group was dramatically higher than the treatment group at both 8 d and 32 d. The verbascoside content in the treatment group reached the highest at 20 d, which was 1.75 times of that in the control group (Table 2).

The content of both total phenylethanol glycosides and total cycloartenoid glycosides was low at the early stage of hairy root growth (8 d), and the secondary metabolites gradually accumulated with the extension of incubation time (Table 2).

### 2.6. Effect of Pp-AgNPs on Sucrose Metabolism in R. glutinosa Hairy Roots In Vivo

**Figure 4:**
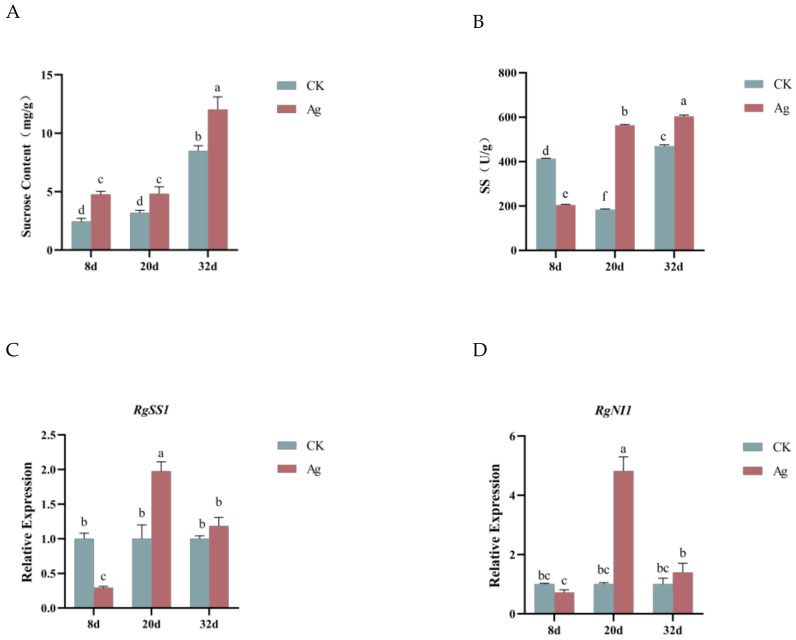
Effect of Pp-AgNPs on sucrose metabolism in *R. glutinosa* hairy roots. (**A**) Sucrose content. (**B**) Activities of sucrose synthase (SS). (**C**) Relative expression of *RgSS1* gene. (**D**) Relative expression of *RgNI1* gene. Different letters represent statistical significance (*p* < 0.05).

The sucrose in the hairy roots gradually accumulated with the extension of incubation time, and the sucrose content in the treatment group was significantly higher than that in the control group after exogenous supplementation of Pp-AgNPs. The sucrose content of the treatment group reached 12.025 mg/g at 32 d, which was 1.42 times higher than that of the control (Figure 4A). The results revealed that Pp-AgNPs enhanced the absorption and storage sucrose in hairy roots.

The metabolism of sucrose in the plants is dependent on the catalysis of sucrose synthase (SS) and neutral invertase (NI). In the present study, we found that RgSS activity was significantly lower in the Pp-AgNPs-treated group as compared to the control group at 8 d, whereas RgSS activity was remarkably higher than the control group at 20 d and 32 d (Figure 4B). The trend of the *RgSS1* gene expression level was basically the same as that of the RgSS activity, with a significant down-regulation of expression at the early stage of growth (8 d) compared with that of the control group, and a dramatic up-regulation of expression at the rapid-growth stage (20 d) and the late-growth stage (32 d) (Figure 4C,D). NI catalyzes irreversibly hydrolysis of sucrose to fructose and glucose. The expression level of the *RgNI1* gene was consistent with that of the *RgSS* gene, with the highest expression of the *RgNI1* gene at 20 d of Pp-AgNPs treatment, which was 1.5-fold of the control.

### 2.7. Expression Analysis of Genes Related to Phytohormone in R. glutinosa Hairy Roots

#### 2.7.1. Expression Analysis of Gibberellin-Related Genes in *R. glutinosa* Hairy Roots

**Figure 5:**
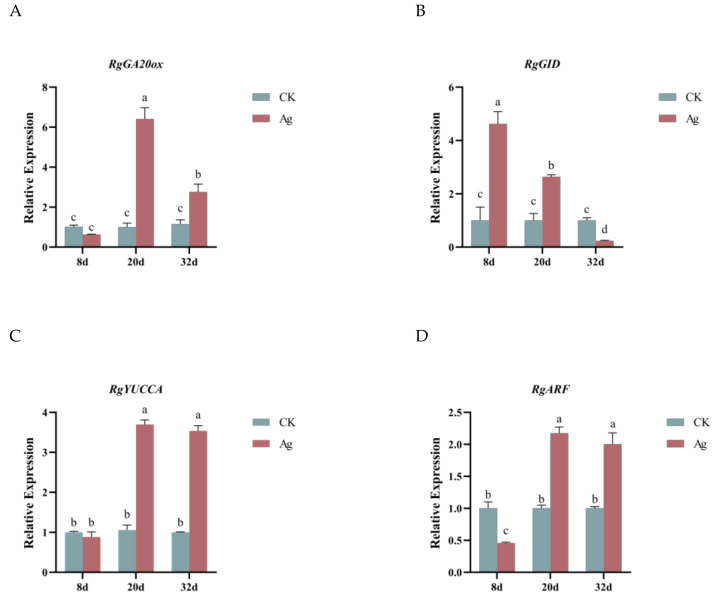
Expression analysis of genes related to GA and IAA in *R. glutinosa* hairy roots. (**A**) Relative expression of *RgGA20ox* gene. (**B**) Relative expression of *RgGID* gene. (**C**) Relative expression of *RgYUCCA* gene. (**D**) Relative expression of *RgARF* gene. Different letters represent statistical significance (*p* < 0.05).

Gibberellin (GA) is an important plant growth-regulating hormone that plays an important role in plant growth and development such as increasing cell elongation, improving biomass, and promoting fruit development. GA20 oxidase (GA20ox) is a key enzyme involved in the GA biosynthesis pathway, catalyzing the conversion of GA12 and GA53 into the active forms GA1 and GA. In *Arabidopsis thaliana*, the silencing of GA20ox-related genes resulted in dwarfed plants, and growth and development were impaired [28]. After Pp-AgNPs treatment, the expression of the *RgGA20ox* gene was not significantly varied between the treated group and the control group in the early growth period (8 d), but was remarkably up-regulated in the rapid-growth period (20 d) and late-growth period (32 d), which were 6.14 and 2.75 times higher than that of the control group, respectively (Figure 5A). The GID protein is a receptor for GA and, together with DELLA proteins, act as major components of the GA signal transduction pathway that promote plant growth by activating the plant’s response to GA. As shown in Figure 5B, the expression of the *RgGID* gene, in the early-growth and rapid-growth stages, was significantly higher than the control group, while in the late-growth stage, its expression was dramatically lower than the control.

We hypothesized that Pp-AgNPs-treated hairy roots showed a dramatic increase in *RgGA20ox* expression accompanied by the up-regulation of *RgGID* expression during their rapid-growth period. This subsequently caused an enhancement in GA content and activated the response of hairy roots to GA, which cooperatively facilitated the growth of hairy roots.

#### 2.7.2. Expression Analysis of Indoleacetic Acid-Related Genes in *R. glutinosa* Hairy Roots

As an essential plant growth hormone, indoleacetic acid (IAA) is involved in plant physiological processes such as tissue differentiation, organogenesis, and apical dominance as well as adaptation to complex environments. *YUCCA* encodes a flavin monooxygenase-like enzyme that catalyzes the formation of IAA from indole pyruvate. The expression level of *RgYUCCA* gene did not alter significantly during the early growth period of hairy roots (8 d), and was 3.57 and 3.4 times higher than the control during the rapid-growth period (20 d) and late-growth period (32 d), respectively (Figure 5C). The expression of the *RgARF* gene was slightly down-regulated compared to the control at 8 d, and was dramatically up-regulated at the rapid-growth stage (20 d) and late-growth stage (20 d, 32 d), which were 2.17 and 2.00 times higher than the control, respectively (Figure 5D). As a whole, the *RgYUCCA* and *RgARF* genes had similar expression patterns. These results indicated that the expression of key genes for IAA biosynthesis and signal transduction in *R. glutinosa* hairy roots was improved after the addition of Pp-AgNPs, which was able to affect the biosynthesis and transport of IAA, and then prompted the growth and development of hairy roots.

### 2.8. Analysis of EST-SSR Genetic Characterization of R. glutinosa

**Figure 6:**
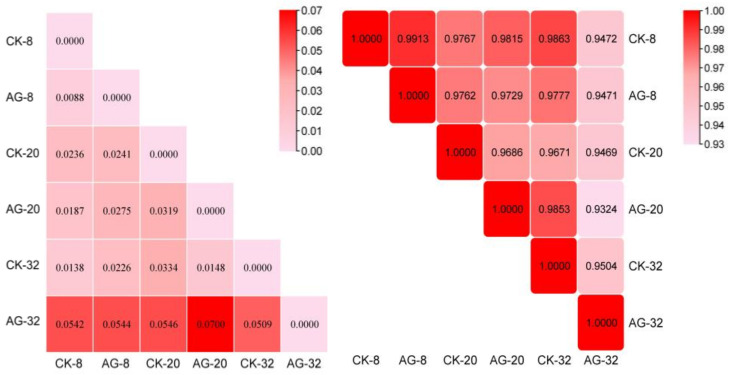
Genetic similarity and genetic distance analysis.

The nine pairs of EST-SSR primers were selected and a total of 37 clear bands was amplified, of which 7 bands exhibited polymorphism, with the percentage of polymorphic bands being 18.92%. The effective alleles (Ne) of different samples ranged from 1.1458 to 1.3674, and the average was 1.3195; Neiʼs genetic diversity index (H) was from 0.2041 to 0.2583, with an average of 0.2315; the Shannon index (I) varied from 0.3140 to 0.3897, and the average was 0.3551 (Figure 6). The index of genetic variation among the samples was 0.0832. In summary, the low genetic diversity of different samples indicated that there was no genetic variation in the hairy roots before and after the treatment, and the application of Pp-AgNPs did not alter the stability of genetic material in the hairy roots.

The genetic distances between the six samples treated with Pp-AgNPs and the control were 0.0088–0.07. The larger value of the genetic distance demonstrated the lower genetic similarity between the two samples. The genetic distance between AG-8 and CK-8 was 0.0088, which was the smallest genetic distance between two samples, indicating that the probability of genetic variation between individual samples was small under different treatment conditions.

## 3. Discussion

The unique physical and chemical properties of AgNPs, a novel material, make it possible to influence the growth and development of plants [29]. Gupta et al. [30] found that AgNPs were able to promote shoot and root growth of rice seedlings under in vitro conditions by enhancing chlorophyll content and reducing ROS levels. The medium containing AgNPs stimulated the formation of *Lavandula angustifolia* Mill. shoots and increased the weight of the plants [15]. In addition, AgNPs were capable of dramatically improving the dry and fresh weight of *Datura metel* L. hairy roots [20]. However, AgNPs may also have inhibitory effects on plant growth due to its concentration, shape, and plant species, etc. For instance, a higher concentration (15.4 mg/L) of AgNPs led to a reduction in *Physalis peruviana* L. seedling size and root system under in vitro culture conditions [31]. Seedlings were exposed to different concentrations (0, 0.1, 0.3, and 0.5 mg/L) of AgNPs for 10 d. The fresh weight of shoots and roots of *Lupinus termis* Forssk. was obviously reduced in the presence of AgNPs above 300 mg/L [32]. In this study, it was found that treatment of *R. glutinosa* hairy roots with 2 mg/L of Pp-AgNPs was effective in promoting the accumulation of hairy root biomass, but the growth of *R. glutinosa* hairy roots was inhibited when the concentration of Pp-AgNPs exceeded 10 mg/L.

Plants respond to adversity stress by regulating secondary metabolism to enhance their adaptability and resistance. As a stress substance, an elicitor can induce plant defense responses and trigger the synthesis of a series of chemicals by modulating signal molecules and signal transduction mechanisms in plants [33]. As a result, secondary metabolic pathways are altered, and the biosynthesis of specific plant secondary product associated with plant defense systems is adapted. AgNPs, as a novel elicitor, can improve plant growth and development, and influence the accumulation of secondary metabolites within a certain concentration range [18]. The effect of AgNPs on plant secondary metabolites has two sides. On the one hand, appropriate amounts of AgNPs promote the biosynthesis of plant secondary metabolites. For example, the induction of AgNPs (5 mg/L) in suspension cultures of *Momordica charantia* L. cells exhibited higher contents of flavonol, hydroxybenzoic acid, and hydroxycinnamic acid [34]. An amount of 100 mg/L AgNPs treatment enhanced the yield of total tropane alkaloids in the hairy roots of *Hyoscyamus muticus* L. [35]. AgNP-β-CDs induced a 1.8-fold increase in total tanshinone content in the hairy roots of *Salvia miltiorrhiza* Bunge. after 7 d of treatment. On the other hand, high concentrations of AgNPs may inhibit the accumulation of plant secondary metabolites [36]. Begum et al. [37] reported that the total flavonoid content of *Fagonia indica* callus was reduced after induction of 250 μg/mL biosynthesized AgNPs for 10 d. The content of secondary metabolites such as anthocyanins and carotenoids in *Calendula officinalis* L. was dramatically decreased after 1.2 mmol/L AgNPs treatment [38]. The impact on the biosynthesis of secondary metabolites varied due to different plant species, different parts, and different AgNPs treatments. In this study, 2 mg/L of Pp-AgNPs was applied to induce the hairy roots of *R. glutinosa*. After 20 d of induction, there was a significant increase in the verbascoside, total phenylethanol glycosides, and total cycloartenoid glycosides in the hairy roots. These results illustrated that the suitable concentrations of Pp-AgNPs promoted the yield of secondary metabolites. Therefore, Pp-AgNPs can be used as a novel elicitor to produce secondary metabolites in *R. glutinosa* hairy roots.

Sucrose accumulation was a protective mechanism against adversity stress in plants. Jha A B et al. [39] reported that sucrose synthase (SS) activity was remarkably enhanced in rice seedlings responding to heavy metal arsenic stress, which provided an adequate carbon source for energy expenditure in the seedlings. We found that the exogenous addition of Pp-AgNPs increased the sucrose content in the treatment group, which may be attributed to the fact that the hairy roots prompted the uptake and accumulation of sucrose in response to the stress of Pp-AgNPs. SS and NI are key enzymes that enable sucrose to enter various metabolic pathways, and SS and NI are capable of converting sucrose into fructose and glucose [40]. Fructose and glucose produced from the breakdown of sucrose by SS and NI can be used as signaling molecules to regulate the activity of some gene promoters or transcription factors, which in turn regulate the vital activities of plants. The high activity of SS in plants is associated with the rapid growth of plant tissues. The SS has two main activities in plants. One is sucrose anabolic activity, which catalyzes the synthesis of sucrose and UDP from UDPG and fructose, and the other is sucrose catabolic activity, which catalyzes the production of fructose and UDPG from sucrose and UDP. In antisense *SS* transgenic hairy roots of *Centella asiatica* (L.) Urb., the *SS* gene expression level was decreased by 75%, and the biomass of the hairy roots was also significantly reduced, which indicated that *SS* was able to affect the growth of hairy roots by regulating carbon metabolism [41]. The sucrose catabolic activity of SS increased when plants were subjected to stress [42]. In the present study, we found that SS enzyme activity, *RgSS* and *RgNI1* gene expression levels in *R. glutinosa* hairy roots, decreased at the beginning of the induction period, whereas remarkably increased during the rapid-growth stage (20 d) and late-growth stage (32 d). We speculated that Pp-AgNPs induction caused stress to the *R. glutinosa* hairy roots, leading to an enhancement in the expression levels of *RgSS* and *RgNI1* genes, which prompted the catabolic activity of SS and facilitated the growth of hairy roots through the regulation of sucrose metabolism.

AgNPs stress generally disturbs the balance between ROS production and elimination in plant cells, causing an increase in ROS accumulation and thus triggering oxidative stress. Nair et al. [43] found that exposure of rice seedlings to 0.5 mg/L of AgNPs resulted in a dramatic elevation in H_2_O_2_. In this study, the H_2_O_2_ content in the hairy roots of *R. glutinosa* was also remarkably elevated after induction by Pp-AgNPs. In order to avoid the adverse impacts of ROS, a set of antioxidant enzyme defense systems such as SOD, POD, CAT, etc. were initiated in plant cells. The activities of these antioxidant enzymes were enhanced after AgNPs stress to protect plant cells against oxidative stress. POD, SOD, and CAT enzyme activities were increased in AgNPs-treated *Ricinus communis* L. seedlings [44]. The activities of SOD, CAT, ascorbate peroxidase (APX), and glutathione reductase (GR) were significantly up-regulated in AgNP-stressed potato plantlets [45]. SOD and CAT activities in *S. miltiorrhiza* hairy roots showed a gradual enhancement after AgNP-β-CD treatment [36]. We found that Pp-AgNPs treatment dramatically elevated the activities of CAT, SOD, and POD in the hairy roots of *R. glutinosa*. The increase in the activities of these antioxidant enzymes suggested that the *R. glutinosa* hairy roots adapted to the stress of AgNPs by improving the antioxidant defense system.

## 4. Materials and Methods

### 4.1. Materials

The hairy roots were induced by *Agrobacterium rhizogenes* ACCC10060 from the leaves of the ‘Beijing No.3′ variety of *R. glutinosa*, and were cultured in 1/2 MS solid medium. Under aseptic conditions, the young leaves of the sterile seedlings of *R. glutinosa* were selected, cut into small pieces of 0.5 cm^2^, and then inoculated on hormone-free MS solid medium. The explants were pre-cultured for 2–3 d, then removed and immersed in *A. rhizogenes* ACCC10060 with the OD_600_ value of 0.6, and infiltrated at 28 °C, 120 rpm for 10 min. The explants were removed and dried up, transferred to MS + AS 200 μmol/L solid medium, and incubated under dark condition. After 3–4 d, the infiltrated explants were transferred to 1/2 MS liquid medium containing 600 mg/L Amp and washed twice at 120 rpm for 15 min. After the explants were blotted dry with sterile filter paper, they were placed on 1/2 MS + 600 mg/L Amp solid medium and cultured at 25 °C in the dark to induce hairy roots, which were succeeded every 7 d for 4–5 times consecutively. Pp-AgNPs were synthesized and characterized according to the method previously reported by Zhu et al. [26].

### 4.2. The Effect of Pp-AgNPs on the Growth of R. glutinosa Hairy Roots

#### 4.2.1. The Identification of an Optimal Concentration of Pp-AgNPs

A series of Pp-AgNPs concentrations (0, 1, 2, 4, 8, 10, 20, 30, 40, 50 mg/L) were added to the hairy roots of *R. glutinosa*, which had been cultured for 7 d. The culture was continued for 20 d for harvesting, and the growth status of the hairy roots was observed and the fresh weights of the hairy roots were measured, so as to screen out the optimal culture concentration.

#### 4.2.2. Establishment of Hairy Roots Growth Curve

An amount of 0.2 g of hairy roots were transferred to a conical flask containing 50 mL of 1/2 liquid medium (with 30 g/L sucrose, pH 5.8) and incubated in a shaker in dark (25 °C, 120 r/min). Following a period of 5–7 d, an appropriate quantity of Pp-AgNPs was introduced to the medium, reaching a final concentration of 2 mg/L, and incubation under dark conditions was conducted. The growth curves of the hairy roots were plotted using fresh weight (g) as the vertical coordinate and incubation time (d) as the horizontal coordinate. After treatment with Pp-AgNPs, the hairy roots were rinsed 3 times repeatedly with distilled water, and filter paper was used to absorb the surface moisture, after which the hairy roots were dried in an oven at 50 °C until constant weight. Dry and fresh weights of hairy roots were measured according to the growth curves at one time point each in the slow-growth, logarithmic, and stable periods.

### 4.3. Detection of Ag Content in Hairy Roots

The content of Ag was determined by inductively coupled plasma mass spectrometry (ICP-MS, iCAP RQ, Thermo Fisher, Lenexa, KS, USA). The standard curve was established through a series of dilutions, beginning with a standard solution of Ag at a concentration of 1000 μg/mL. Approximately 0.2 g of hairy roots samples were added into a digestion tube containing 5 mL concentrated HNO_3_ and were digested at 105 °C for 30 min using a microwave digestion system (MARS 2, CEM, Charlotte, NC, USA). The samples were passed through a 0.22 μm filter and diluted to 50 mL with deionized water.

### 4.4. Measurement of Color

The color changes of hairy roots in the control and treated groups were quantified using a colorimeter (CM-5, Konica Minolta, Tokyo, Japan). The light source was D65, the starting and stopping wavelengths were 350–750 nm, and the standard deviation of ΔE* ab was ≤0.04. Luminance (L*) represents brightness, with a larger L* value indicating greater brightness. (a*) represents red-green phase, with a negative value indicating green and a positive value indicating red. (b*) represents yellow-blue phase, with a negative value indicating blue and a positive value indicating yellow.

### 4.5. Antioxidant Enzyme Activity Assay

Antioxidant enzymes were extracted at 4 °C from 0.5 g of fresh tissue. The samples were homogenized with 5 mL of extraction buffer containing 0.2 mmol/L EDTA and 0.05 M PBS with a pH of 7.8. The extracts were subjected to centrifugation at 12,000 rpm for a period of 10 min, after which the resulting supernatant was utilized for the determination of SOD, CAT, and POD activities. The activities of the antioxidant enzymes were quantified using an Enzyme Activity Assay Kit (Nanjing Jiancheng Bioengineering Institute, Nanjing, China). All analyses were repeated 3 times.

### 4.6. Measurement of H_2_O_2_ Content

The content of H_2_O_2_ was assayed as described by Zhu [46]. An amount of 0.2 g of *R. glutinosa* hairy roots was weighed, ground into a fine powder with liquid nitrogen, and then added with 2 mL of PBS (pH = 7.0) to create a homogeneous slurry, and then centrifuged at 12,000 rpm for 10 min, after which the supernatant was collected. The supernatant was divided into two portions, one for the determination of H_2_O_2_ content and the other for the measurement of protein concentration using the Bradford method, which was then brought into the equation to calculate the H_2_O_2_ content. The H_2_O_2_ concentration was determined using a hydrogen peroxide assay kit (Nanjing Jiancheng Bioengineering Institute, Nanjing, China), according to the manufacturer’s instructions. The content of H_2_O_2_ was calculated using the following equation:

$$H_{2}O_{2}\;(\mathrm{mmol}/\mathrm{gprot})=\frac{A_{\mathrm{sa}}-A_{\mathrm{ck}}}{A_{\mathrm{st}}-A_{\mathrm{ck}}}\;\times \;C_{\mathrm{st}}÷C_{\mathrm{pr}}$$

A_sa_ = absorbance A value of the sample to be measured.

A_ck_ = absorbance A value of the control group.

A_st_ = absorbance A value of standard solution.

C_st_ = concentration of H_2_O_2_ standard solution (163 mmol/L).

C_pr_ = protein concentration of plant sample (gprot/L, prot refers to protein).

### 4.7. Determination of Sucrose Content and Sucrose Synthase Activity

An amount of 1 g of hairy roots was ground into powder. The sugar extraction was conducted in accordance with the methodology proposed by Li et al. [47]. Sucrose content and sucrose synthase activity were determined by Solarbio assay kit (Beijing Solarbio Science & Technology Co., Ltd., Beijing, China).

### 4.8. Detection of Secondary Metabolite Content

High performance liquid chromatography (HPLC) was employed for the determination of verbascoside in *R. glutinosa* hairy roots. An amount of 1 g of *R. glutinosa* hairy roots powder was weighed and 70% methanol was added. The solution was sonicated for 45 min, filtered, and the volume was adjusted to a 50 mL vial well. An amount of 2.5 mL was measured and 70% methanol was added as the test solution. The mobile phase was acetonitrile/acetic acid (16:84). The column was ZORBAX SB-Aq (4.6 mm × 250 mm, 5-micron) at 30 °C with a flow rate of 1 mL/min and a wavelength of 334 nm.

The content of total iridoid glycosides and total phenylethanoid glycoside were determined by ultraviolet spectrophotometry. The standard curve was drawn with catalpol as the representative of total iridoid glycosides, and verbascoside as the representative of total phenylethanoid glycoside. The vertical coordinate was the absorbance value at 465 nm and 332 nm, respectively, and the horizontal coordinate was the mass concentration of the respective compounds.

### 4.9. Quantitative Real-Time PCR

RNA was extracted using the RNeasy Plant Mini Kit (Qiagen, Hilden, Germany). cDNA was made using the RevertAid First Strand cDNA Synthesis Kit (Fermentas, Vilnius, Lithuania). The primers were designed using Primer Premier v5.0. The primer sequences were listed on Table S1. The qRT-PCR analysis was then performed on the QuantStudio5 Real-Time PCR System (Thermo Fisher, Lenexa, KS, USA). The targeted DNA amplification procedure was set as follows: 95 °C for 3 min and 35 cycles (95 °C for 10 s, 56 °C for 20 s, 72 °C for 30 s), and the melting curve was from 65 to 95 °C. Gene expression levels were normalized to the internal reference gene *TIP41* and measured by the 2^−ΔΔct^ method. All reactions were replicated three times.

### 4.10. Analysis of Genetic Diversity of R. glutinosa Hairy Roots

The DNA was extracted from the *R. glutinosa* hairy roots samples using an Ezup column genomic DNA extraction kit. The samples were amplified using 9 pre-screened EST-SSR primers for *R. glutinosa* (Table S2). The PCR system was as follows: 1 μL of DNA, 1 μL each of upstream and downstream primers, 10 μL of mix, and 7 μL of ddH2O. PCR conditions: 95 °C for 3 min; 95 °C for 30 s, 72 °C for 30 s, 35 cycles; 72 °C for 10 min. The PCR products were electrophoresed on a 2% agarose gel. The gel was observed and imaged using a ChemiDoc XRS system (Bio-Rad, Boulder, CO, USA). The bands with polymorphism were labeled using software to help with labeling, and a 0/1 matrix was exported as raw data to calculate the ratio of polymorphic bands. The Popgene v1.32 was used to analyze the genetic diversity and distance of different *R. glutinosa* hairy roots samples.

### 4.11. Statistical Analysis

The data were analyzed using the statistical software package SPSS 18.0, with the results expressed as mean ± standard deviation. The Duncan multiple range test was employed for the comparison of the groups. A *p*-value of less than 0.05 was considered statistically significant.

## 5. Conclusions

**Figure 7:**
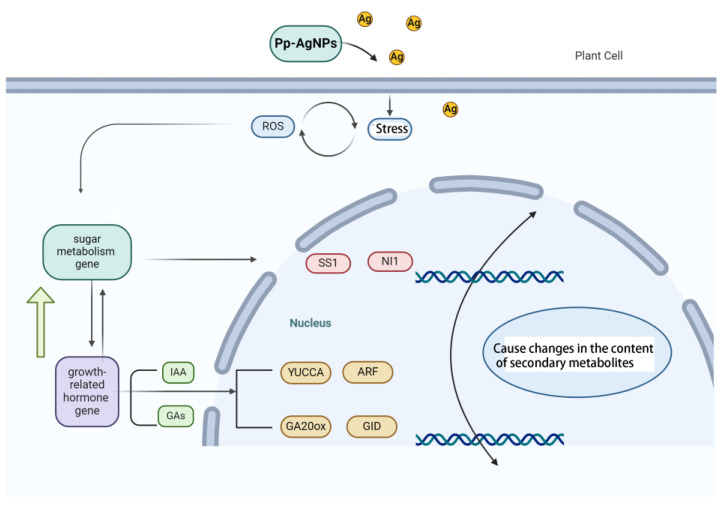
Pp-AgNPs on the growth of hairy roots of *R. glutinosa* and secondary metabolism.

In this study, we investigated the induction effects of Pp-AgNPs in the hairy root culture of the medicinal plant *R. glutinosa*, and analyzed the mechanism of Pp-AgNPs in facilitating the production of secondary metabolites of *R. glutinosa*, which provided a basis for the in vitro cultivation, utilization, and promotion of *R. glutinosa* secondary metabolites. The dry weight, fresh weight, verbascoside, total iridoid glycosides, and total phenylethanol glycoside contents of *R. glutinosa* hairy roots were all higher in the treated group than in the control group after addition of 2 mg/L Pp-AgNPs for 20 d. Pp-AgNPs were able to prompt the antioxidant enzymes’ activities by inducing the production of ROS in *R. glutinosa* hairy roots (Figure 7). The growth-promoting effect of Pp-AgNPs may be accomplished by increasing sucrose metabolism, and regulating the synthesis and signal transduction of GA and IAA (Figure 7). Meanwhile, genetic characterization revealed that Pp-AgNPs treatment did not alter the genetic material and stability of hairy roots. In conclusion, the appropriate concentration of Pp-AgNPs can be used as an effective elicitor to improve the biomass and secondary metabolites content in *R. glutinosa* hairy roots.

## Figures and Tables

**Table 1:** Accumulation of Ag in the hairy roots of *R. glutinosa*.

Time/d	CK Ag (μg/g)	AgNPs Ag (μg/g)
8	0.08 ± 0.00 d	151.82 ± 0.08 a
20	0.12 ± 0.00 d	111.75 ± 0.22 c
32	0.21 ± 0.00 d	144.28 ± 0.24 b

Different letters represent statistical significance (*p* < 0.05).

**Table 2:** The content of secondary metabolites in *R. glutinosa* hairy roots treated with Pp-AgNPs.

Time (d)	Verbascoside (%)		Total Phenylethanoid Glycosides (%)		Total Iridoid Glycosides (%)	
	CK	AgNPs	CK	AgNPs	CK	AgNPs
8	3.32 ± 0.00 b	2.99 ± 0.01 c	1.03 ± 0.01 e	1.09 ± 0.06 e	0.99 ± 0.10 e	0.78 ± 0.04 f
20	2.37 ± 0.00 d	4.16 ± 0.01 a	2.61 ± 0.01 d	3.95 ± 0.00 a	6.45 ± 0.13 d	9.32 ± 0.10 a
32	1.29 ± 0.00 e	1.10 ± 0.00 f	3.75 ± 0.02 b	3.43 ± 0.02 c	8.39 ± 0.14 c	8.89 ± 0.19 b

Different letters represent statistical significance (*p* < 0.05).

## Data Availability

The original contributions presented in this study are included in the article; further inquiries can be directed to the corresponding author.

## Supplementary Materials

The following supporting information can be downloaded at: https://www.mdpi.com/article/10.3390/ijms252313088/s1.
