# Supplementary material for: Green Synthesized Silver Nanoparticles Induced Accumulation of Biomass and Secondary Metabolites in Hairy Roots of Rehmannia glutinosa
Source: Int J Mol Sci. 2024 Dec 5;25(23):13088. doi: 10.3390/ijms252313088 (PMC11642862; doi:10.3390/ijms252313088)
Supplement: Supplementary file 1 [file ijms-25-13088-s001.zip › ijms-3314494-supplementary.pdf]

Table S1. List of gene primers used for gene expression studies.

Name	Upstream primer （5′-3′）	Downstream primer（5′-3′）
RgTIP41	TGGCTCAGAGTTGATGGAGTGCT	CTCTCCAGCAGCTTTCTCGGAGA
RgGA20ox	TTCGGCTGAATCACTACCC	ATGCCTTACACTCTTGAACCTC
RgGID	CCCGTAATCGTGTTCTTCCAT	CCAAAACCTCCACATTCTCCT
RgYUCCA	GATGGTGTTTGCCACAGG	AGCCCAGCACAATAAAGC
RgARF	TTCAGAAGTGTTACAGGCAAGTT	GGATTAGGATCTGTCAGGAGGC
RgSS1	GCGAAGTGCTAAGGCTGATGAA	CAGTAGTGTCCGTTGCTGTGAAATA
RgNI1	CCGAATACCGACTGCTGACAAT	AGCTTCAAGTCTTTCACCGTTC

Table S2. List of gene primers used for EST-SSR.

Name	Upstream primer （5′-3′）	Downstream primer（5′-3′）
SSR1	AGAGAGGTGGCTGTGGA	ACATCATCAGGAAACCTCCG
SSR2	CTGTTTTGACCCGACAGGTT	AGATTCCGATTTCGATGACG
SSR3	ACCATTCACACCCCCACATT	TTACGGCGGATCTACGAATC
SSR4	TGAGCAAACCTCAAGCAACA	GAAGTGCCTTTGCGTCTTTC
SSR5	GCAACGGTTCAGGAAAATGT	TCGAGCTCATCATCTTCTTCAC
SSR6	GAGACCTCTTGAGGGTGCAA	AAGCAGGAATGAGTACGCT
SSR7	GAACAAACTCGTGTGGGGTT	CTGCAACAATCAACCAATCG
SSR8	AATTGTCCCAAACCCTAGCC	TATCCCAACCATAAACCCCA
SSR9	CCCAAAACTCCACTGCCTAA	GGTCTTCTTCGAGTGGCTTG
